# Development and validation of a score to predict mortality in ICU patients with sepsis: a multicenter retrospective study

**DOI:** 10.1186/s12967-021-03005-y

**Published:** 2021-07-29

**Authors:** Jie Weng, Ruonan Hou, Xiaoming Zhou, Zhe Xu, Zhiliang Zhou, Peng Wang, Liang Wang, Chan Chen, Jinyu Wu, Zhiyi Wang

**Affiliations:** 1 Department of General Practice, The Second Affiliated Hospital and Yuying Children’s Hospital of Wenzhou Medical University, Wenzhou, 325027 China; 2 Department of Emergency Intensive Care Unit, The Second Affiliated Hospital and Yuying Children’s Hospital of Wenzhou Medical University, Wenzhou, 325027 China; 3 Department of Public Health, Robbins College of Health and Human Sciences, Baylor University, Waco, TX USA; 4 Department of Geriatric Medicine, The First Affiliated Hospital, Wenzhou Medical University, Wenzhou, 325000 China; 5 Institute of Genomic Medicine, Wenzhou Medical University, Wenzhou, 325000 China; 6 Institute of Bioscaffold Transplantation and Immunology, School of Basic Medical Sciences, Wenzhou Medical University, Wenzhou, 325035 China; 7 Center for Health Assessment, Wenzhou Medical University, Wenzhou, China

**Keywords:** Sepsis, Intensive care unit, Mortality prediction score

## Abstract

**Background:**

Early and accurate identification of septic patients at high risk for ICU mortality can help clinicians make optimal clinical decisions and improve the patients’ outcomes. This study aimed to develop and validate (internally and externally) a mortality prediction score for sepsis following admission in the ICU.

**Methods:**

We extracted data retrospectively regarding adult septic patients from one teaching hospital in Wenzhou, China and a large multi-center critical care database from the USA. Demographic data, vital signs, laboratory values, comorbidities, and clinical outcomes were collected. The primary outcome was ICU mortality. Through multivariable logistic regression, a mortality prediction score for sepsis was developed and validated.

**Results:**

Four thousand two hundred and thirty six patients in the development cohort and 8359 patients in three validation cohorts. The Prediction of Sepsis Mortality in ICU (POSMI) score included age ≥ 50 years, temperature < 37 °C, Respiratory rate > 35 breaths/min, MAP ≤ 50 mmHg, SpO2 < 90%, albumin ≤ 2 g/dL, bilirubin ≥ 0.8 mg/dL, lactate ≥ 4.2 mmol/L, BUN ≥ 21 mg/dL, mechanical ventilation, hepatic failure and metastatic cancer. In addition, the area under the receiver operating characteristic curve (AUC) for the development cohort was 0.831 (95% CI, 0.813–0.850) while the AUCs ranged from 0.798 to 0.829 in the three validation cohorts. Moreover, the POSMI score had a higher AUC than both the SOFA and APACHE IV scores. Notably, the Hosmer–Lemeshow (H–L) goodness-of-fit test results and calibration curves suggested good calibration in the development and validation cohorts. Additionally, the POSMI score still exhibited excellent discrimination and calibration following sensitivity analysis. With regard to clinical usefulness, the decision curve analysis (DCA) of POSMI showed a higher net benefit than SOFA and APACHE IV in the development cohort.

**Conclusion:**

POSMI was validated to be an effective tool for predicting mortality in ICU patients with sepsis.

**Supplementary Information:**

The online version contains supplementary material available at 10.1186/s12967-021-03005-y.

## Background

Sepsis remains the leading cause of death in patients admitted to the Intensive Care Unit (ICU), worldwide and is therefore a great challenge to clinicians [1–3]. According to guidelines by the Surviving Sepsis Campaign, timely diagnosis and identification of patients at risk is recommended to provide aggressive early intervention and improve the prognosis of septic patients [4]. Therefore, early recognition of septic patients at high risk is essential for reducing fatality. Additionally, it is necessary to build a reliable predictive system for clinicians to improve the patients’ outcomes given that sepsis is a complex, heterogeneous disease associated with high morbidity and mortality. Moreover, trustworthy clinical management strategies can be provided for critically ill patients, with the assistance of various predictive systems.

Prediction of mortality in sepsis continues to be the main focus of critical care medicine. In addition, accurate clinical risk prediction models can objectively estimate disease severity and stratify patients according to the risk of death. They may also alert clinicians and allow for timely identification of high-risk populations that require aggressive management and intervention. Consequently, several predictive systems have been developed to date, including the Acute Physiology and Chronic Health Evaluation (APACHE) II and IV scores [5, 6], Simplified Acute Physiology Score (SAPS) II and III [7, 8], Sequential Organ Failure Assessment (SOFA) score [4], Predisposition, Insult/Infection, Response and Organ Dysfunction (PIRO) [9] and the Charlson comorbidity index [10]. Although these scores have been applied widely in a variety of patient groups, especially those who are critically ill, they may exhibit poor sensitivity or/and specificity and low reproducibility when applied to some specific diseases [11]. Notably, there are currently no risk prediction systems available specifically for septic patients.

Therefore, the present study attempted to develop and validate (both internally and externally) a mortality prediction score based on clinical and laboratory data from more than 200 hospitals, to estimate mortality in ICU patients with sepsis. The study also compared the prediction performance of the score with that of the APACHE IV and SOFA scores. It was hypothesized that the risk prediction score developed by the study would more accurately predict the risk for sepsis than both the APACHE IV and SOFA scores, hence being more beneficial to septic patients.

## Materials and methods

### Data sources

This was a multicenter, retrospective, observational study that was conducted using data from the eICU Collaborative Research Database which is a large multi-center critical care database containing information on 139,367 patients from 335 ICUs in 208 hospitals across the USA, in 2014 and 2015 [12, 13]. The study also obtained patient clinical data from the Second Affiliated Hospital and Yuying Children’s Hospital of Wenzhou Medical University, Wenzhou, China, which had over 2000 beds. Data on septic patients admitted to the hospital between January 1, 2010 and September 31, 2020 was obtained through the electronic medical record management system. The study was approved by the Ethics Committee of the Second Affiliated Hospital and Yuying Children’s Hospital of Wenzhou Medical University.

### Participants

Participants were enrolled based on the definition of sepsis-3 i.e. a known or suspected infection plus SOFA > 2 points for organ dysfunction [14, 15]. In addition, the first ICU admission was selected for septic patients admitted to the ICU more than once. The study however excluded patients who were younger than 18 years of age. Considering the different medical care levels in different regions, we divided the patients into three groups (Midwest, West and South) according to the hospital locations in the USA. Septic patients from the Midwest were used as the development cohort because of the largest sample size. Patients from West and South were used as external validation sets. And, data from the Chinese ICU was acted as another external validation set.

### Variables

The Structured Query Language (SQL) with pgAdmin 4 (version 4.30) was used to extract data from the eICU database. The study retrospectively collected the following data: (1) demographic information including age, sex, race, height and weight; (2) site of infection, including pulmonary, renal/urinary tract infection (UTI), cutaneous/soft tissue, Gastrointestinal (GI), gynecologic, others, and unknown; (3) APACHE IV and SOFA scores on the day of ICU admission; (4) vital signs including temperature, heart rate, respiratory rate, systolic pressure, diastolic pressure, mean arterial pressure (MAP) and oxygen saturation levels at the first records after ICU admission; (5) laboratory data, including albumin, bicarbonate, bilirubin, creatinine, glucose, hematocrit, hemoglobin, lactate, platelet, blood urea nitrogen (BUN), white blood cell (WBC) and alanine transaminase (ALT) within 24 h of ICU admission; (6) comorbidities including Acquired Immunodeficiency Syndrome (AIDS), hepatic failure, lymphoma, metastatic cancer, leukemia, immunosuppression, and cirrhosis. For laboratory data recorded more than once, values associated with the most severe form of sepsis were employed. The proportion of missing values was less than 10% across all the variables.

### Endpoints

The main outcome of the present study was ICU mortality. Survival following admission to the ICU was clearly recorded in eICU database. On the other hand, electronic medical records were used to retrieve information on patients who survived following admission in the ICU of the Second Affiliated Hospital and Yuying Children’s Hospital of Wenzhou Medical University.

### Statistical analysis

The Shapiro–Wilk test was used to examine whether the data was normally distributed. Categorical variables were described by frequency (percentages) and mean (SD) or median (interquartile range) for continuous variables, as appropriate. In addition, non-normal continuous variables were compared using the Wilcoxon rank-sum test while the Student’s t test was employed for the normally distributed data. Moreover, categorical variables were analyzed using the chi-squared test or Fisher’s exact test, accordingly.

The primary outcome for the study was ICU mortality. Therefore, univariate logistic regression analyses were conducted to identify the unadjusted association between potential predictors and ICU mortality. For the ICU mortality model, the backward stepdown logistic regression based on the smallest Akaike Information Criterion (AIC) value was selected to confirm the independent risk variables for ICU mortality [16]. Additionally, multicollinearity of variables was examined using the Variance Inflation Factor (VIF) for each predictive variable and a VIF ≥ 5 indicated multicollinearity among variables.

Thereafter, the above continuous independent predictor variables were transformed into categorical variables based on quartiles then all the categorical variables (including AIDS, hepatic failure and metastatic cancer) were subjected to multivariable logistic regression to identify the final predictor variables in the prediction scoring system. The study developed this scoring system to predict mortality in septic patients and named it, Prediction of Sepsis Mortality in the ICU (POSMI). POSMI was developed by allocating an integer or half an integer score, which was calculated by dividing the regression coefficient of each predictor variable with the smallest regression coefficient. The sum of each predictor variable score yielded a total score for each individual and this total score was included in the final regression model. In addition, the model’s discrimination for ICU mortality was examined using the area under the receiver operating characteristic curve (AUC) and calibration was conducted using calibration curves and the Hosmer–Lemeshow (H–L) goodness-of-fit test. Moreover, the DeLong’s non-parametric method was used to compare the two AUC values with an equal sample size [17]. Following recommendations by Hosmer and Lemeshow, an AUC ≥ 0.7 indicated an acceptable discrimination while an AUC ≥ 0.8 showed excellent discrimination. Furthermore, Integrated Discrimination Improvement (IDI) was used to evaluate improvement in model performance [18] and the 95% Confidence Intervals (CIs) were calculated using non-parametric bootstrapping. In order to assess the clinical utility of the POSMI score, Decision Curve Analysis (DCA) was performed to compare the net benefit of the POSMI, APACHE IV and SOFA scores in the prediction of ICU mortality, at different threshold probabilities.

Given that missing data could have influenced the results to some extent, the multiple imputation technique using chained equations was employed in order to minimize bias and maintain the power of the study before data analysis. The “mice” package in R was used to implement this method. Additionally, sensitivity analyses were conducted to evaluate the influence of missing value filling. All the statistical analyses were conducted using R (version 3.6.1) and a *p* value < 0.05 was considered to be statistically significant.

## Results

### Populations

Ten thousand seven hundred and fifty four septic patients from the eICU database met the inclusion criteria. Based on geographical locations in the USA, 4236 patients were from the Midwest, 3185 from the West, 2934 from the South, 386 from the Northeast and 13 were from unknown locations. In addition, the study consecutively collected a total of 1878 sepsis cases from the Second Affiliated Hospital and Yuying Children’s Hospital of Wenzhou Medical University, Wenzhou, China, between January 2010 and September 2020. Notably, septic patients from the Midwest, who had the largest sample size, were used as the development cohort. The median age in the development cohort was 69 years (range, 57 to 80 years) and 53% of the patients were male. Additionally, the infection sites most frequently associated with sepsis were pulmonary (41%), renal/UTI (23%), GI (13%), unknown (10%), cutaneous/soft tissue (8%) and others (4%). The ICU mortality and hospital mortality rates were 11.8%, and 19.1%, respectively. On the other hand, patients from the West of USA and Wenzhou, China were used as the validation set, named as the West and Wenzhou validation cohorts. Moreover, patients from the Northeast and unknown regions were grouped into the South region due to the small sample sizes and used as another validation set, named as the South validation cohort. Table 1 shows the demographic and clinical characteristics of the development and validation cohorts.

**Table 1:** Demographic and clinical characteristics of the patients with sepsis following ICU admission

Variables	Midwest development cohort (n = 4236); n (%)	West validation cohort (n = 3185)		South validation cohort (n = 3333)		Wenzhou validation cohort (n = 1878)	
		n (%)	*P* value*	n (%)	*P* value*	n (%)	*P* value*
Demographics and social history							
Age, median (IQR, years)	69 (57, 80)	68 (56, 79)	0.027	69 (58, 80)	0.939	69 (57.25, 80)	0.949
Male sex, n (%)	2252 (53)	1650 (52)	0.26	1716 (52)	0.164	1020 (54)	0.422
Race, n (%)			< 0.001		< 0.001		
African American	411 (10)	107 (3)		629 (19)			
Asian	53 (1)	82 (3)		44 (1)			
Caucasian	3426 (81)	2495 (78)		2277 (68)			
Hispanic	54 (1)	183 (6)		219 (7)			
Native American	23 (1)	79 (2)		13 (0)			
Other/unknown	269 (6)	239 (8)		151 (5)			
Height, median (IQR, cm)	170 (161.8, 177.8)	170 (162, 177.8)	0.481	167.6 (160, 177.8)	0.035	170 (160, 177.8)	0.796
Weight, median (IQR)	77.1 (63.5, 95)	77.6 (64.4, 96.5)	0.244	77 (63.5, 94.2)	0.697	76.9 (63.4, 93.5)	0.207
Infection site, n (%)			0.014		< 0.001		< 0.001
Pulmonary	1745 (41)	1212 (38)		929 (28)		725 (39)	
Renal/UTI	985 (23)	770 (24)		862 (26)		389 (21)	
Cutaneous/soft tissue	337 (8)	280 (9)		231 (7)		140 (7)	
GI	544 (13)	468 (15)		384 (12)		250 (13)	
Gynecologic	9 (0)	14 (0)		12 (0)		2 (0)	
Other	173 (4)	111 (3)		420 (13)		137 (7)	
Unknown	443 (10)	330 (10)		495 (15)		235 (13)	
Ventilation, n (%)	1411 (33)	1142 (36)	0.024	1110 (33)	1	643 (34)	0.497
Severity score							
SOFA, median (IQR)	5 (3, 7)	5 (3, 7)	< 0.001	5 (3, 7)	0.004	5 (3, 7)	0.001
APACHE IV, median (IQR, Kg)	67 (53, 84)	71 (56, 90)	< 0.001	71 (57, 89)	< 0.001	71 (55, 90.75)	< 0.001
Vital signs							
Temperature, median (IQR)	37.4 (36.9, 38.1)	37.3 (36.9, 38.1)	0.787	37.3 (36.9, 38.1)	0.140	37.4 (36.9, 38.1)	0.673
Heart rate, median (IQR)	109 (94, 125)	112 (97, 128)	< 0.001	110 (96, 126)	0.020	111 (96, 127)	0.004
Respiratory rate, median (IQR)	29 (24, 35)	29 (25, 35)	0.004	29 (24, 35)	0.011	29 (24, 35)	0.036
Systolic pressure, median (IQR)	85 (75, 96)	84 (73, 95)	< 0.001	83 (72, 95)	< 0.001	84 (74, 96)	0.012
Diastolic pressure, median (IQR)	45 (37, 52)	44 (36, 52)	0.018	44 (36, 51)	0.006	44 (36, 52)	0.093
MAP, median (IQR, mmHg)	60 (52, 68)	59 (51, 67)	0.003	59 (51, 66)	< 0.001	59 (51, 67)	0.059
SpO2, median (IQR)	92 (89, 95)	92 (87, 95)	< 0.001	92 (88, 95)	0.112	92 (88, 95)	0.195
Laboratory tests							
Albumin, median (IQR)	2.6 (2.1, 3)	2.5 (2.1, 3)	0.008	2.5 (2.1, 3)	0.039	2.5 (2.1, 3)	0.028
Bicarbonate, median (IQR)	21 (17, 24)	20 (17, 24)	< 0.001	20 (17, 24)	< 0.001	20 (16.1, 23)	< 0.001
Bilirubin, median (IQR)	0.8 (0.5, 1.4)	0.8 (0.5, 1.5)	0.060	0.8 (0.5, 1.4)	0.658	0.8 (0.5, 1.4)	0.442
Creatinine, median (IQR)	1.67 (1.1, 2.81)	1.73 (1.11, 2.91)	0.143	1.7 (1.1, 2.83)	0.438	1.77 (1.11, 2.95)	0.114
Glucose, median (IQR)	107 (89, 136)	108 (88, 137)	0.771	108 (88, 136)	0.523	107 (88, 135)	0.283
Hematocrit, median (IQR)	31 (26.4, 35.5)	30.7 (26.1, 35.2)	0.051	30.8 (26, 35.4)	0.070	30.5 (26.6, 35.48)	0.381
Hemoglobin, median (IQR)	10.1 (8.6, 11.7)	10 (8.4, 11.6)	0.038	10 (8.4, 11.6)	0.033	10 (8.6, 11.7)	0.509
Lactate, median (IQR)	2.4 (1.5, 4.2)	2.6 (1.6, 4.6)	< 0.001	2.6 (1.6, 4.4)	< 0.001	2.7 (1.6, 4.68)	< 0.001
Platelet, median (IQR)	157 (106, 226)	157 (105, 222)	0.489	160 (102, 224)	0.419	155 (105, 231)	0.915
BUN, median (IQR)	33 (21, 52)	34 (22, 53)	0.122	34 (22, 53)	0.054	35 (22, 56)	0.002
WBC, median (IQR)	15.62 (10.4, 21.91)	15.8 (10.7, 22.5)	0.121	16 (10.6, 22.8)	0.063	15.8 (10.9, 22.3)	0.103
ALT, median (IQR)	28 (18, 56)	30 (18, 60)	0.141	30 (18, 59)	0.072	30 (18, 58)	0.165
Morbidities							
Dialysis, n (%)	205 (5)	168 (5)	0.426	170 (5)	0.641	91 (5)	1
AIDS, n (%)	10 (0)	17 (1)	0.059	9 (0)	0.951	2 (0)	0.365
Hepatic failure, n (%)	75 (2)	80 (3)	0.033	77 (2)	0.114	56 (3)	0.003
Lymphoma, n (%)	34 (1)	30 (1)	0.606	31 (1)	0.638	24 (1)	0.104
Metastatic cancer, (%)	138 (3)	114 (4)	0.489	119 (4)	0.496	1806(96)	0.287
Leukemia, n (%)	69 (2)	59 (2)	0.521	73 (2)	0.089	28 (1)	0.744
Immunosuppression, n (%)	213 (5)	192 (6)	0.068	200 (6)	0.072	103 (5)	0.496
Cirrhosis, n (%)	123 (3)	107 (3)	0.292	113 (3)	0.253	58 (3)	0.756
Outcome							
ICU mortality, n (%)	500 (12)	471 (15)	< 0.001	467 (14)	0.005	268 (14)	0.008
Hospital mortality, n (%)	808 (19)	688 (22)	0.008	724 (22)	0.005	398 (21)	0.059
Length of ICU stay, median (IQR)	53 (29, 101)	55 (29, 113)	0.172	53 (28, 105)	0.95	54 (28, 100.75)	0.736

Continuous data are presented as median (interquartile range), whereas categorical data are presented as frequency (percentage)

^*^p values compare the development cohort to each of the three validation cohorts using Wilcoxon Mann–Whitney test or exact Fisher test depending on whether the variable is continuous or categorical

### Predictors of ICU mortality

Univariate logistic regression was used to test for the potential risk factors that would predict ICU mortality. Most of the variables were associated with ICU mortality (Additional file 1: Table S1). However, the study performed the backward stepdown multivariate logistic regression analysis based on the smallest AIC value in order to determine the independent risk factors. The results revealed fourteen independent risk factors including; age, temperature, heart rate, respiratory rate, MAP, SpO_2_, mechanical ventilation, albumin, bilirubin, lactate, BUN, AIDS, hepatic failure and metastatic cancer (Additional file 1: Table S2). Thereafter, continuous variables were converted into categorical variables based on the quartiles in order to further validate the above continuous independent predictor variables and for practical purposes. Multivariable logistic regression analysis identified age ≥ 50 years, temperature < 37 °C, respiratory rate > 35 breaths/min, MAP ≤ 50 mmHg, SpO2 < 90%, albumin ≤ 2 g/dL, bilirubin ≥ 0.8 mg/dL, lactate ≥ 4.2 mmol/L, BUN ≥ 21 mg/dL, mechanical ventilation, hepatic failure and metastatic cancer (Additional file 1: Table S3).

### The POSMI score

Twelve variables were used to create the POSMI score and each prognostic variable was assigned a score (Table 2). The POSMI score for each patient was derived by obtaining a sum of the points corresponding to prognostic factors, whose scores ranged from 0 to 25. In addition, septic patients in the Midwest development cohort were divided into four categories according to each POSMI score distribution. These included the low risk (0–6 points) category which had 1.2% ICU mortality, moderate risk (7–10 points) which had 6.3% ICU mortality, high risk (11–15 points) which had 23.3% ICU mortality and very high risk (> 7 points) which had 66.9% ICU mortality (Table 3).

**Table 2:** Risk factors for predictive model for ICU mortality in the midwest development cohort (n = 4236)

Variable	β	OR (95%CI)^a^	*P*-value	Point^b^
Age, years				
≥ 50 to < 60	0.7901	2.203 (1.294–3.899)	0.005	2
≥ 60 to < 75	1.0021	2.724 (1.641–4.724)	< 0.001	2.5
≥ 75	1.2261	3.407 (2.050–5.923)	< 0.001	3
Temperature < 37 °C	0.7332	2.081 (1.546–2.811)	< 0.001	2
Respiratory rate, breaths/min				
≥ 30 to < 35	0.6111	1.842 (1.312–2.600)	< 0.001	1.5
≥ 35	0.5886	1.801 (1.292–2.526)	< 0.001	1.5
MAP ≤ 50, mmHg	0.8744	2.397 (1.673–3.481)	< 0.001	2
SpO2 < 90%	0.9508	2.587 (1.926–3.502)	< 0.001	2
Ventilation	1.2014	3.325 (2.665–4.156)	< 0.001	3
Albumin < 2, g/dL	0.6289	1.875 (1.343–2.639)	< 0.001	1.5
Bilirubin, mg/dL				
≥ 0.8 to < 1.4	0.5366	1.710 (1.202–2.456)	0.003	1
≥ 1.4	0.7915	2.206 (1.559–3.158)	< 0.001	2
Lactate, mmol/L				
≥ 2.5 to < 4.2	0.4558	1.577 (1.117–2.241)	0.010	1
≥ 4.2	0.9476	2.579 (1.869–3.595)	< 0.001	2
BUN, mg/dL				
≥ 21 to < 33	0.4779	1.612 (1.117–2.349)	0.012	1
≥ 33 to < 52	0.4119	1.509 (1.049–2.192)	0.028	1
≥ 52	1.0401	2.829 (2.006–4.043)	< 0.001	2.5
Hepatic failure	1.0094	2.744 (1.468–4.993)	0.001	2.5
Metastatic cancer	0.5894	1.802 (1.051–2.997)	0.027	1
Total score				0–25

*OR *odds ratio

^a^ICU mortality odds ratio

^b^Assignment of points to risk factors was based on a linear transformation of the corresponding β regression coefficient. The coefficient of each variable was divided by 0.4119 (the smallest absolute β value, corresponding to BUN ≥ 33 to < 52, mg/dL) and allocated an integer or an half integer score for each variable

**Table 3:** Risk of ICU mortality in the development and validation cohorts according to risk stratification

Risk stratification	n (%)	Predicted ICU mortality % (95% CI)	Actual ICU mortality %
Midwest development cohort			
Low	1126 (26.6)	1.3 (1.2–1.3)	1.2
Moderate	1836 (43.3)	6.1 (5.8–6.4)	6.3
High	1105 (26.1)	22.7 (21.7–23.0)	23.3
Very high	169 (4.0)	66.8 (65.1–67.9)	66.9
West validation cohort			
Low	789 (24.8)	1.8 (1.7–1.8)	1.8
Moderate	1302 (40.9)	7.3 (7.1–7.4)	7.3
High	908 (28.5)	25.0 (24.5–26.6)	26.2
Very high	186 (5.8)	66.3 (64.6–68.0)	66.7
South validation cohort			
Low	816 (24.5)	1.4 (1.3–1.4)	1.3
Moderate	1424 (42.7)	6.4 (6.4–6.5)	6.5
High	915 (27.5)	27.6 (27.1–28.4)	28.3
Very high	178 (5.3)	58.6 (57.6–59.6)	59.0
Wenzhou validation cohort			
Low	462 (24.6)	2.4 (2.3–2.5)	2.4
Moderate	746 (39.7)	8.2 (7.7–8.3)	8.2
High	564 (30.0)	22.7 (21.8–23.4)	23.4
Very high	106 (5.7)	60.0 (57.7–62.7)	60.4

The risk category was calculated by adding the points for each of the following risk factors. The prognostic index was categorized in four groups: low risk (0–6 points), moderate risk (> 6– ≤ 10 points), high risk (> 10– ≤ 15 points), and very high risk (> 15 points)

### Risk stratification

Classification of the Midwest development cohort based on the POSMI score resulted in 1126 (26.6%) patients in the low-risk class, 1,836 (43.3%) in the moderate-risk group, 1105 (26.1%) in the high-risk category and 169 (4.0%) in the very high-risk class (Table 3). Classification results for the West, South and Wenzhou validation cohorts were similar to those of the development cohort. In the West, South and Wenzhou validation cohorts, 24.8%, 24.5%, and 24.6% of the patients, respectively, were assigned to the low-risk class; 40.9%, 42.7% and 39.7%, respectively, fell under the moderate-risk group; 28.5%, 27.5% and 30.0%, respectively, were classified into the high-risk category and 5.8%, 5.3% and 5.7%, respectively, were assigned to the very high-risk class (Table 3). Moreover, patients in the three validation cohorts showed ICU mortality rates similar to those in the development cohort, in the four risk classifications (Table 3). Furthermore, the ICU mortality predicted by the POSMI score was very close to that of actual ICU mortality in the four different risk levels (Table 3).

### Validation of the POSMI score

**Fig. 1:**
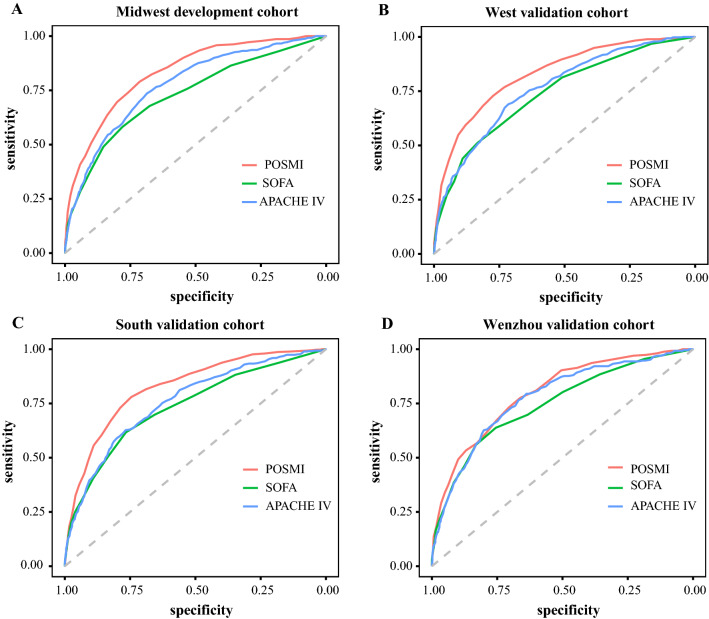
Receiver operating characteristic curves of POSMI, SOFA and APACHE IV scores in predicting ICU mortality in the development and validation cohorts. Receiver operating characteristic curves of the three scores in predicting mortality in the (**A**) Midwest development cohort, (**B**) West validation cohort, (**C**) South validation cohort and (**D**) Wenzhou validation cohort

Performance of the POSMI score was compared to that of the SOFA and APACHE IV scores in predicting ICU mortality in septic patients. The AUC value for the POSMI score was 0.831 (95% CI, 0.813–0.850) and was significantly higher than that of the SOFA score which was 0.728 (95 CI, 0.703–0.754) and the APACHE IV score which was 0.773 (95% CI, 0.752–0.795), in the development cohort (Fig. 1A; Table 4). This indicated that the POSMI score had better discrimination than both the SOFA and APACHE IV scores. Similarly, the AUC values for the POSMI score in the West and South validation cohorts were more than 0.8 and were also significantly higher than those of both the SOFA and APACHE IV scores. The results therefore showed that the POSMI score had excellent discrimination in predicting mortality in ICU patients with sepsis (Fig. 1B, C; Table 4). In the Wenzhou validation cohort, the AUC value of the POSMI score was 0.798 (95% CI, 0.769–0.826) and was higher than that of the SOFA score which was 0.747 (95% CI, 0.714–0.780) and the APACHE IV score which was 0.777 (95% CI, 0.747–0.807). However, no significant differences in AUC were obtained between POSMI and APACHE IV (Fig. 1D; Table 4).

**Table 4:** Comparison of models in predicting the ICU mortality of sepsis

	Predictive model	AUC	*P* value	HL Chi-square	*P* value	IDI	*P* value
Midwest development cohort	POSMI	0.831 (0.813–0.850)		10.963	0.2038		
	SOFA	0.728 (0.703–0.754)	< 0.001			0.102 (0.082–0.123)	< 0.001
	APACHE IV	0.773 (0.752–0.795)	< 0.001			0.081 (0.060–0.102)	< 0.001
West validation cohort	POSMI	0.829 (0.809–0.049)		3.0918	0.9285		
	SOFA	0.741 (0.716–0.766)	< 0.001			0.108 (0.085–0.131)	< 0.001
	APACHE IV	0.763 (0.740–0.786)	< 0.001			0.095 (0.073–0.117)	< 0.001
South validation cohort	A-SIMP	0.825 (0.805–0.845)		10.888	0.2081		
	SOFA	0.736 (0.740–0.786)	< 0.001			0.084 (0.062–0.105)	< 0.001
	APACH IV	0.758 (0.734–0.782)	< 0.001			0.077 (0.057–0.098)	< 0.001
Wenzhou validation cohort	POSMI	0.798 (0.769–0.826)		13.135	0.1073		
	SOFA	0.747 (0.714–0.780)	0.005			0.042 (0.014–0.069)	0.003
	APACHE IV	0.777 (0.747–0.807)	0.208			0.035 (0.001–0.060)	0.007

*AUC* area under curve, *IDI *integrated discrimination improvement, *HL* Hosmer–Lemeshow

**Fig. 2:**
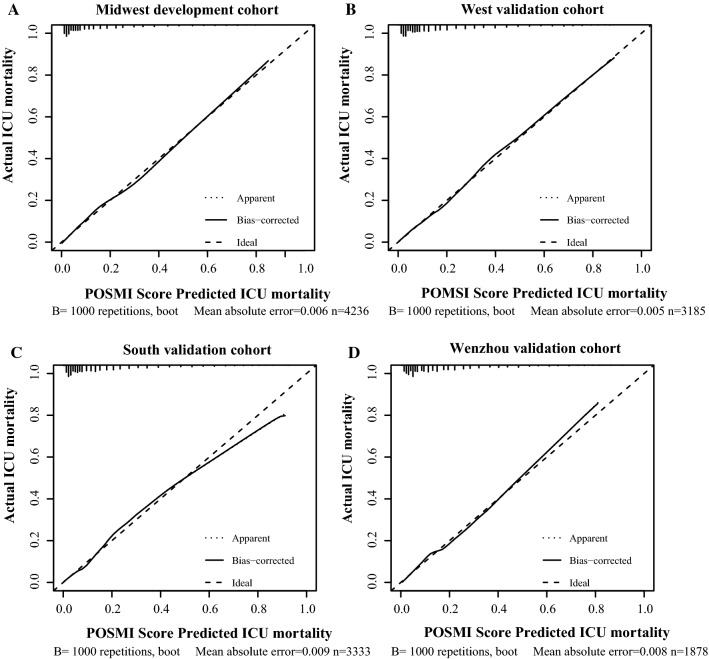
Calibration curves constructed through the bootstrap approach in the development and validation cohorts. The predicted ICU mortality was in agreement with the actual ICU mortality in the (**A**) Midwest development cohort, (**B**) West validation cohort, (**C**) South validation cohort and (**D**) Wenzhou validation cohort (**D**)

Additionally, the accuracy of the POSMI score was assessed using calibration curves and the H–L Chi-square test, in the development and validation cohorts. The bias-corrected curve, generated through a bootstrap method, showed a slight deviation from the reference line although the predicted ICU mortality was still in good agreement with the actual ICU mortality (Fig. 2). Moreover, the H–L Chi-square test showed that the POSMI score had good calibration in the Midwest development cohort (HL Chi-square = 10.963; p = 0.204). Good calibration was also confirmed in the West (HL Chi-square = 3.092; p = 0.929), South (HL Chi-square = 10.888; p = 0.208) and Wenzhou validation cohorts (HL Chi-square = 13.135; p = 0.107) as shown in Table 4. In addition, there was a significant increase in the IDI of the POSMI score compared to that of the SOFA and APACHE IV scores in the development and validation cohorts. This suggested that the POSMI score could improve significantly in prediction performance (Table 4). Furthermore, excellent discrimination and calibration were still observed in the sensitivity analyses when missing values were excluded, in the development and validation cohorts. In addition, the AUCs for predicting ICU mortality in the Midwest, West, South and Wenzhou cohorts were 0.794 (95% CI, 0.767–0.822), 0.818 (95% CI, 0.789–0.847), 0.835 (95% CI, 0.811–0.859) and 0.826 (95% CI, 0.790–0.862), respectively. On the other hand, the H–L goodness-of-fit test results were 13.761 (p = 0.088), 2.657 (p = 0.954), 1.008 (p = 0.201) and 7.405(p = 0.493), respectively.

### Net benefit of using the POSMI score

**Fig. 3:**
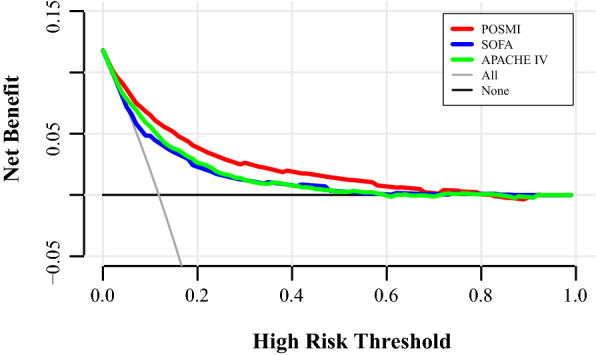
The DCA curve of medical intervention in patients with the POSMI, SOFA, and APACHE IV scores in the Midwest development cohort

Decision curve analysis (Fig. 3) showed that POSMI had a positive net benefit at a predicted threshold probability between 1 and 80% compared to treating septic patients as if they would all have died or they would all have survived (i.e., treat-all or treat-none strategies). The estimates of net benefits from using the POSMI score at different probability thresholds are provided in Table 5 (more estimates of net benefits are shown in Table S4). When the predicted threshold probability was 1% to 60% for the SOFA score and 1–80% for the APACHE IV score, the net benefits were positive in both scores (Table 5). With regard to clinical use, medical treatment aided by POSMI had more net benefit than using both the SOFA and APACHE IV scores when the predicted threshold probability was between 1 and 80% (Fig. 3; Table 5).

**Table 5:** Net benefit of using the POSMI score, APACHE IV and SOFA compared to treating sepsis assuming all of them will die during the ICU stay

Threshold probability	Net benefit				Advantage of using A-SIMP score
	Treat all	POSMI score	APACHE IV	SOFA	Difference in net benefit^a^
0.01	0.924	0.93	0.925	0.924	0.006
0.02	0.847	0.863	0.851	0.847	0.016
0.03	0.769	0.813	0.777	0.769	0.044
0.04	0.689	0.777	0.709	0.689	0.088
0.05	0.607	0.731	0.665	0.608	0.124
0.1	0.169	0.553	0.474	0.409	0.384
0.15	− 0.319	0.433	0.321	0.296	0.752
0.2	− 0.869	0.328	0.222	0.195	1.197
0.25	− 1.492	0.257	0.149	0.138	1.749
0.3	− 2.203	0.223	0.103	0.103	2.426
0.35	− 3.025	0.182	0.076	0.089	3.207
0.4	− 3.983	0.162	0.064	0.067	4.145
0.45	− 5.116	0.139	0.04	0.065	5.255
0.5	− 6.475	0.114	0.024	0.026	6.589
0.55	− 8.136	0.096	0.02	0.013	8.232
0.6	− 10.212	0.059	− 0.005	0.004	10.271
0.65	− 12.881	0.047	− 0.004	0.01	12.928
0.7	− 16.441	0.013	− 0.011	0.005	16.454
0.75	− 21.424	0.03	0.006	0.01	21.454
0.8	− 28.898	0.004	0.004	0.008	28.902

^a^Compare with treat all

## Discussion

The present study involved 12,631 patients admitted with sepsis to more than 300 ICUs in over 200 hospitals. The study developed and validated (both internally and externally) a POSMI score for predicting the risk of ICU mortality. Although some of the predictor variables in the risk score have been reported previously, there is a limited number of tools for predicting the risk of mortality in septic patients [19–21]. Notably, the novel POSMI score developed by the study had a number of advantages. The score could easily be implemented based on the available common variables and had good calibration as well as discrimination for ICU mortality in septic patients in both the development and validation cohorts. Additionally, the discrimination and IDI of the POSMI score were significantly higher than those of the APACHE IV and SOFA scores (discrimination of the POSMI score was similar to that of the APACHE IV score in Wenzhou validation cohort). The score may therefore be ideal for guiding decision-making in clinical practice for the management of septic patients. Moreover, the POSMI score showed comparable or better discrimination for predicting ICU mortality in sepsis, compared to other predictive scoring systems in sepsis and critically ill patients. Such include prediction of mortality in sepsis (AUC, 0.68–0.75) [20, 22–24], prediction of ICU mortality in surgical patients (AUC, 0.72) [25], prediction of mortality in the critically ill with sepsis using the SOFA score (AUC, 0.77) [26] and prediction of mortality in an academic cardiac intensive care unit using the APACHE IV score (AUC, 0.82) [27]. Considering the high morbidity and mortality rates associated with sepsis, it is necessary to establish a risk score for clinicians to accurately predict and evaluate the outcomes of septic patients. This will also be important in clinical decision-making.

Body temperature is a main area of focus in studies on sepsis [28]. For instance, two recent studies on body temperature and sepsis showed that hyperpyrexia was associated with poor prognosis in septic patients [29, 30]. In addition, a randomized controlled trial demonstrated that fever control by external cooling, significantly reduced early mortality in septic shock [31]. Additionally, most studies showed that hypothermia was associated with a higher mortality in septic patients [32–34]. According to a previous study, the occurrence of fever in sepsis may be associated with better survival [35]. However, the present study found an association between an admission body temperature below 37 °C and the risk of ICU mortality although body temperature alone was not sufficiently predictive of the severity of illness. In addition to body temperature, the study showed that respiratory rate and blood pressure were also predictors of poor outcomes in patients with sepsis. It is noteworthy that the two have been adopted as predictors in many critical illness prediction scoring systems, such as qSOFA [20] as well as the APACHE II and IV scores [5, 6]. Moreover, heart rate was not independently associated with mortality from sepsis in the study. Nonetheless, variability in heart rate was associated with mortality from sepsis in some studies previously reported [36–38]. Consequently, heart rate at admission was not incorporated in the prediction model for convenience of implementation and practical implications. The POSMI score also included age, SpO2, mechanical ventilation, hepatic failure, metastatic cancer and clinical laboratory values. Patients with low SpO2 or mechanical ventilation may have already developed sepsis-induced acute respiratory failure/Acute Respiratory Distress Syndrome (ARDS) which is associated with high case-fatality [2, 39, 40]. Furthermore, the clinical laboratory variables incorporated in the present prediction model are plausible risk factors for sepsis. Notably, low albumin, high bilirubin and BUN reflect acute and/or chronic damage of the liver and kidney, which are both strong and independent risk factors of prognosis in critical illness [41]. Additionally, high serum lactate was proven to be significantly associated with mortality in patients with sepsis [42–44]. All these factors could therefore provide important prognostic information for the prediction model.

Performance of the model in this study was evaluated based on discrimination and calibration through statistical analysis and graphical methods. The AUC for the development and validation cohorts ranged from 0.798 to 0.831, reflecting the excellent ability of the model to discriminate ICU mortality in patients. Additionally, the H–L goodness-of-fit test results and calibration curves suggested that the predicted ICU mortality was similar to the actual ICU mortality, indicating that the prediction model was well calibrated. Moreover, the study validated the calibration of the prediction model at four risk levels (low-, moderate-, high- and very high risk). Expectedly, the ICU mortality predicted by the model was almost consistent with the actual ICU mortality. In addition, the IDI of POSMI in the development and validation cohorts were all significantly higher than those of the APACHE IV and SOFA score, suggesting that the prediction model was superior to both the APACHE IV and SOFA scores. The study also used cohorts with no missing values to conduct sensitivity analysis. Although the multiple imputation approach was used, the POSMI score still maintained excellent discrimination and calibration. With regard to clinical benefit, patients could get more net benefit from using the POSMI score.

Although a high- or a very high-risk score does not directly influence treatment decision-making, it may be useful in making objective prognoses and recommendations for clinicians as well as patients and their families. Nevertheless, further studies are required to confirm the clinical application of the POSMI score. In addition, clinical trials on sepsis may benefit from using the POSMI score as an inclusion and exclusion criterion. For instance, very high-risk patients, where therapeutic measures may not bring clinical benefits because of the severity of disease and low-risk patients whose event rate may be too low to warrant inclusion, may be excluded to optimize the study design. Furthermore, the POSMI score could facilitate patient stratification in clinical studies.

The present study had a number of strengths. First, the POSMI score had excellent model performance in the development and external validation cohorts. The POSMI score was also relatively easy to calculate and all the variables could easily be obtained. In addition, the development and validation cohorts were from hospitals of different sizes (most hospitals from the eICU database are small and medium-sized while the Wenzhou validation cohort came from a large-sized hospital), making it possible to use the model in other hospitals or countries. Nonetheless, the study had a few limitations. First, this was a retrospective cohort study and although we adjusted for many potential confounders, the possibility of residual confounders remains, and the POSMI score was only validated in USA and China, further validation is needed to determine whether our prediction model is applied to other locations or countries. Second, information on the time from onset of illness to hospital admission was missing. Third, the reported ICU mortality was all-cause mortality, the cause of death was not available in the cohorts. Finally, there was no information about treatment in preventing the ICU mortality. Most notably, the baseline differences between the study populations (different continents), ICU practices, study dates (2 years vs. 10 years, one of which includes the COVID-19 pandemic which is likely to have influenced the ICU data collected during that time) were not addressed in present study. Hence, more prospective studies are therefore needed to validate these findings.

## Conclusions

In conclusion, the present study developed and validated a simple risk score, POSMI score, which is valuable in predicting mortality in septic patients admitted to the ICU. The POSMI score is superior to the SOFA and APACHE IV scores in present study. We anticipate it will be most useful for risk stratification and decision-making.

## Supplementary Information

**Additional file 1: Table S1**. The prognostic factors of ICU mortality in univariate analysis. **Table S2**. The prognostic factors of ICU mortality in multivariate logistic analysis. **Table S3**. The independent prognostic factors of ICU mortality. **Table S4**. Net benefit of using the POSMI score, APACHE IV and SOFA compared to managing sepsis assuming all of them will die during the ICU stay.

## Data Availability

The datasets used and/or analysed during the current study are available from the corresponding author on reasonable request.

## Abbreviations

ICU: Intensive care unit
APHACHE: Acute physiology and chronic health evaluation
SAPS: Simplified acute physiology score
SOFA: Sequential organ failure assessment
PIRO: Predisposition, insult/infection, response and organ dysfunction
SQL: Structured query language
UTI: Urinary tract infection
MAP: Mean arterial pressure
BUN: Blood urea nitrogen
WBC: White blood cell
ALT: Alanine transaminase
ARDS: Acquired immunodeficiency syndrome
AIC: Akaike information criterion
VIF: Variance inflation factor
POSMI: Prediction of sepsis mortality in the ICU
ROC: Receiver operating characteristic curve
H–L: Hosmer–Lemeshow
IDI: Integrated discrimination improvement
CI: Confidence interval
DCA: Decision curve analysis
SpO2: Oxyhemoglobin saturation
ARDS: Acute respiratory distress syndrome
