# Supplementary material for: Development and validation of a score to predict mortality in ICU patients with sepsis: a multicenter retrospective study
Source: J Transl Med. 2021 Jul 29;19:322. doi: 10.1186/s12967-021-03005-y (PMC8319895; doi:10.1186/s12967-021-03005-y)
Supplement: Supplementary file 1 — Additional file 1: Table S1. The prognostic factors of ICU mortality in univariate analysis. Table S2. The prognostic factors of ICU mortality in multivariate logistic analysis. Table S3. The independent prognostic factors of ICU mortality. Table S4. Net benefit of using the POSMI score, APACHE IV and SOFA compared to managing sepsis assuming all of them will die during the ICU stay. [file 12967_2021_3005_MOESM1_ESM.docx]

**Supplementary**

Table 1 The prognostic factors of ICU mortality in univariate analysis

| Variables | Survivors | Non-survivors | *P*-value | β | Unadjusted OR (95%CI) |
| --- | --- | --- | --- | --- | --- |
| Age | 68 (57, 79) | 72 (61, 83) | < 0.001 | 0.0160 | 1.016 (1.009-1.022) |
| Male sex, | 1986 (53) | 266 (53) | 0.986 | 0.0016 | 1.001 (0.830-1.208) |
| Height | 170 (162, 177.8) | 167.6 (160, 177.2) | 0.118 | -0.0058 | 0.994 (0.987- 1.001) |
| Weight | 77.2 (63.9, 94.8) | 75.72 (62.25, 96.12) | 0.177 | -0.0024 | 0.997 (0.994-1.001) |
| Temperature | 37.4 (37, 38.1) | 37.2 (36.7, 38.1) | < 0.001 | -0.2962 | 0.743 (0.667-0.826) |
| Heart rate | 108 (94, 124) | 117.5 (99, 134) | < 0.001 | 0.0140 | 1.014 (1.010-1.018) |
| Respiratory rate | 28 (24, 34) | 32 (28, 38) | < 0.001 | 0.0430 | 1.044 (1.033-1.054) |
| Systolic pressure | 86 (76, 97) | 75 (63, 89) | < 0.001 | -0.0367 | 0.963 (0.958-0.969) |
| Diastolic pressure | 45 (38, 53) | 38 (30, 47) | < 0.001 | -0.0461 | 0.954 (0.947-0.962) |
| MAP | 61 (53, 68) | 53 (44, 61) | < 0.001 | -0.0523 | 0.949 (0.941-0.956) |
| SPO_2_ | 92 (89, 95) | 88.5 (78, 93) | < 0.001 | -0.0470 | 0.954 (0.946-0.960) |
| Ventilation | 1117 (30) | 294 (59) | < 0.001 | 1.2078 | 3.346 (2.765-4.055) |
| Albumin | 2.6 (2.2, 3.1) | 2.2 (1.8, 2.7) | < 0.001 | -0.8331 | 0.434 (0.373-0.504) |
| Bicarbonate | 21 (18, 24) | 18 (14, 22) | < 0.001 | -0.1014 | 0.903 (0.887-0.919) |
| Bilirubin | 0.8 (0.5, 1.4) | 1.1 (0.7, 2.3) | < 0.001 | 0.0963 | 1.101 (1.075-1.128) |
| Creatinine | 1.6 (1.08, 2.7) | 2.2 (1.37, 3.6) | < 0.001 | 0.0761 | 1.079 (1.038-1.119) |
| Glucose | 108 (90, 137) | 101 (76, 133) | 0.002 | -0.0034 | 0.996 (0.994-0.998) |
| Hematocrit | 31.1 (26.6, 35.6) | 29.8 (24.9, 34.8) | < 0.001 | -0.0247 | 0.975 (0.962-0.989) |
| Hemoglobin | 10.2 (8.6, 11.7) | 9.7 (8.07, 11.33) | < 0.001 | -0.0863 | 0.917 (0.879-0.956) |
| Lactate | 2.3 (1.41, 3.8) | 4.2 (2.3, 8.2) | < 0.001 | 0.2283 | 1.256 (1.223-1.290) |
| Platelet | 159 (109, 226) | 143 (72.75, 222) | 0.001 | -0.0015 | 0.998 (0.997-0.999) |
| BUN | 32 (20, 50) | 43 (29, 62.25) | < 0.001 | 0.0098 | 1.009 (1.007-1.012) |
| WBC | 15.5 (10.4, 21.6) | 17.16 (10.4, 24.9) | 0.007 | 0.0058 | 1.005 (1.001-1.010) |
| ALT | 27 (17, 52) | 41 (23, 109) | < 0.001 | 0.0003 | 1.000 (1.000-1.000) |
| Dialysis | 179 (5) | 26 (5) | 0.689 | 0.0861 | 1.089 (0.699 - 1.632) |
| AIDS | 8 (0) | 2 (0) | 0.429 | 0.6267 | 1.871 (0.281-7.494) |
| Hepatic failure | 52 (1) | 23 (5) | < 0.001 | 1.2284 | 3.416 (2.035-5.561) |
| Lymphoma | 27 (1) | 7 (1) | 0.118 | 0.6680 | 1.950 (0.778-4.262) |
| Metastatic cancer | 113 (3) | 25 (5) | 0.020 | 0.5232 | 1.687 (1.060-2.584) |
| Leukemia | 58 (2) | 11 (2) | 0.285 | 0.3552 | 1.426 (0.705-2.628) |
| Immunosuppression | 181 (5) | 32 (6) | 0.136 | 0.2948 | 1.342 (0.895-1.951) |
| Cirrhosis | 91 (2) | 32 (6) | < 0.001 | 1.007 | 2.738 (1.785-4.099) |

OR = odds ratio, AOR = Adjusted odds ratio.

SpO2, percutaneous oxygen saturation; BUN, blood urea nitrogen;

Table 2 The prognostic factors of ICU mortality in multivariate logistic analysis

| Variables | β | Adjusted OR (95%CI) | *P*-value |
| --- | --- | --- | --- |
| Age | 0.0226 | 1.022 (1.015-1.030) | <0.001 |
| Temperature | -0.3119 | 0.731 (0.644-0.828) | <0.001 |
| Heart rate | 0.0053 | 1.005 (1.000-1.010) | 0.035 |
| Respiratory rate | 0.0239 | 1.024 (1.010-1.037) | <0.001 |
| MAP | -0.0247 | 0.975 (0.966-0.984) | <0.001 |
| SPO_2_ | -0.0310 | 0.969 (0.961-0.977) | <0.001 |
| Ventilation | 1.1093 | 3.032 (2.431-3.787) | <0.001 |
| Albumin | -0.4520 | 0.636 (0.533-0.757) | <0.001 |
| Bilirubin | 0.0771 | 1.080 (1.049-1.111) | <0.001 |
| Lactate | 0.1495 | 1.161 (1.127-1.197) | <0.001 |
| BUN | 0.0062 | 1.006 (1.002-1.009) | <0.001 |
| AIDS | 1.5050 | 4.504 (0.631-20.526) | 0.075 |
| Hepatic failure | 0.6721 | 1.958 (0.978-3.740) | 0.048 |
| Metastatic cancer | 0.5392 | 1.714 (1.003-2.828) | 0.040 |

OR = odds ratio, AOR = Adjusted odds ratio.

SpO2, percutaneous oxygen saturation; BUN, blood urea nitrogen;

Table 3. The independent prognostic factors of ICU mortality

| Variable |  | β | Adjusted OR (95%CI) | *P*-value |
| --- | --- | --- | --- | --- |
| Age, y |  |  |  |  |
| < 50 |  | Reference |  |  |
| ≥ 50 to < 60 |  | 0.7901 | 2.203 (1.294-3.899) | **0.004** |
| ≥ 60 to < 75 |  | 1.0021 | 2.724 (1.641-4.724) | **0.0001** |
| ≥75 |  | 1.2261 | 3.407 (2.050-5.923) | **<0.001** |
| Temperature, ℃ |  |  |  |  |
| ﹥38 |  | Reference |  |  |
| ≥ 37.5 to < 38 |  | -0.1949 | 0.822 (0.580-1.159) | 0.269 |
| ≥ 37 to < 37.5 |  | 0.0112 | 1.011 (0.735-1.389) | 0.944 |
| < 37 |  | 0.7332 | 2.081 (1.546-2.811) | **<0.001** |
| Heart rate, beats/min |  |  |  |  |
| < 95 |  | Reference |  |  |
| ≥ 95 to < 110 |  | -0.1139 | 0.892 (0.639-1.246) | 0.502 |
| ≥ 110 to <120 |  | 0.0231 | 1.023 (0.727-1.441) | 0.894 |
| ≥ 120 |  | 0.2526 | 1.287 (0.924-1.798) | 0.136 |
| Respiratory rate, breaths/min | | |  |  |
| < 25 |  | Reference |  |  |
| ≥25 to < 30 |  | -0.0288 | 0.971 (0.686-1.378) | 0.871 |
| ≥30 to < 35 |  | 0.6111 | 1.842 (1.312-2.600) | **0.001** |
| ≥ 35 |  | 0.5886 | 1.801 (1.292-2.526) | **0.001** |
| MAP, mmHg |  |  |  |  |
| >70 |  | Reference |  |  |
| ﹥60 to ≤ 70 |  | -0.0932 | 0.910 (0.618-1.353) | 0.640 |
| ﹥50 to ≤ 60 |  | 0.2767 | 1.318 (0.923-1.909) | 0.134 |
| ≤ 50 |  | 0.8744 | 2.397 (1.673-3.481) | **<0.001** |
| SpO2, % |  |  |  |  |
| > 95 |  | Reference |  |  |
| ≥ 90 to 95 |  | 0.0822 | 1.085 (0.769-1.531) | 0.639 |
| ≥ 90 to 92 |  | 0.3200 | 1.377 (0.943-2.001) | 0.094 |
| < 90 |  | 0.9508 | 2.587 (1.926-3.502) | **<0.001** |
| Ventilation |  | 1.2014 | 3.325 (2.665-4.156) | **<0.001** |
| Albumin, g/dL |  |  |  |  |
| > 3 |  | Reference |  |  |
| > 2.5 to ≤ 3 |  | 0.1854 | 1.203 (0.845-1.721) | 0.305 |
| > 2.0 to ≤ 2.5 |  | 0.2707 | 1.310 (0.937-1.846) | 0.117 |
| ≤ 2 |  | 0.6289 | 1.875 (1.343-2.639) | **<0.001** |
| Bilirubin, mg/dL |  |  |  |  |
| < 0.5 |  | Reference |  |  |
| ≥ 0.5 to < 0.8 |  | 0.1672 | 1.181 (0.815-1.725) | 0.381 |
| ≥ 0.8 to < 1.4 |  | 0.5366 | 1.710 (1.202-2.456) | **0.003** |
| ≥ 1.4 |  | 0.7915 | 2.206 (1.559-3.158) | **<0.001** |
| Lactate, mmol/L |  |  |  |  |
| < 1.5 |  | Reference |  |  |
| ≥ 1.5 to < 2.5 |  | 0.0739 | 1.076 (0.739-1.570) | 0.699 |
| ≥ 2.5 to < 4.2 |  | 0.4558 | 1.577 (1.117-2.241) | **0.010** |
| ≥ 4.2 |  | 0.9476 | 2.579 (1.869-3.595) | **<0.001** |
| BUN, mg/dL |  |  |  |  |
| < 21 |  | Reference |  |  |
| ≥ 21 to < 33 |  | 0.4779 | 1.612 (1.117-2.349) | **0.011** |
| ≥ 33 to < 52 |  | 0.4119 | 1.509 (1.049-2.192) | **0.028** |
| ≥ 52 |  | 1.0401 | 2.829 (2.006-4.043) | **<0.001** |
| AIDS |  | 1.6846 | 5.390 (0.695-25.965) | 0.057 |
| Hepatic failure |  | 1.0094 | 2.744 (1.468-4.993) | **0.001** |
| Metastatic cancer |  | 0.5894 | 1.802 (1.051- 2.997) | **0.026** |

Table 4. Net benefit of using the POSMI score, APACHE IV and SOFA compared to managing sepsis assuming all of them will die during the ICU stay.

| Threshold probability | Net benefit | | | | Advantage of using A-SIMP Score |
| --- | --- | --- | --- | --- | --- |
|  | Treat All | A-SIMP Score | APACHE IV | SOFA | Difference in net benefit |
| 0 | 1 | 1 | 1 | 1 | 0 |
| 0.01 | 0.924 | 0.93 | 0.925 | 0.924 | 0.006 |
| 0.02 | 0.847 | 0.863 | 0.851 | 0.847 | 0.016 |
| 0.03 | 0.769 | 0.813 | 0.777 | 0.769 | 0.044 |
| 0.04 | 0.689 | 0.777 | 0.709 | 0.689 | 0.088 |
| 0.05 | 0.607 | 0.731 | 0.665 | 0.608 | 0.124 |
| 0.06 | 0.523 | 0.684 | 0.625 | 0.561 | 0.161 |
| 0.07 | 0.437 | 0.636 | 0.589 | 0.494 | 0.199 |
| 0.08 | 0.35 | 0.608 | 0.544 | 0.453 | 0.258 |
| 0.09 | 0.261 | 0.578 | 0.501 | 0.411 | 0.317 |
| 0.1 | 0.169 | 0.553 | 0.474 | 0.409 | 0.384 |
| 0.11 | 0.076 | 0.516 | 0.435 | 0.379 | 0.44 |
| 0.12 | -0.019 | 0.493 | 0.398 | 0.357 | 0.512 |
| 0.13 | -0.117 | 0.47 | 0.362 | 0.336 | 0.587 |
| 0.14 | -0.217 | 0.454 | 0.338 | 0.314 | 0.671 |
| 0.15 | -0.319 | 0.433 | 0.321 | 0.296 | 0.752 |
| 0.16 | -0.424 | 0.405 | 0.309 | 0.281 | 0.829 |
| 0.17 | -0.531 | 0.388 | 0.286 | 0.265 | 0.919 |
| 0.18 | -0.641 | 0.37 | 0.264 | 0.249 | 1.011 |
| 0.19 | -0.753 | 0.343 | 0.25 | 0.206 | 1.096 |
| 0.2 | -0.869 | 0.328 | 0.222 | 0.195 | 1.197 |
| 0.21 | -0.987 | 0.313 | 0.215 | 0.184 | 1.3 |
| 0.22 | -1.108 | 0.296 | 0.203 | 0.172 | 1.404 |
| 0.23 | -1.233 | 0.283 | 0.187 | 0.16 | 1.516 |
| 0.24 | -1.36 | 0.27 | 0.163 | 0.145 | 1.63 |
| 0.25 | -1.492 | 0.257 | 0.149 | 0.138 | 1.749 |
| 0.26 | -1.626 | 0.244 | 0.141 | 0.13 | 1.87 |
| 0.27 | -1.765 | 0.234 | 0.134 | 0.122 | 1.999 |
| 0.28 | -1.907 | 0.223 | 0.118 | 0.114 | 2.13 |
| 0.29 | -2.053 | 0.211 | 0.119 | 0.108 | 2.264 |
| 0.3 | -2.203 | 0.223 | 0.103 | 0.103 | 2.426 |
| 0.31 | -2.358 | 0.214 | 0.097 | 0.097 | 2.572 |
| 0.32 | -2.517 | 0.205 | 0.088 | 0.092 | 2.722 |
| 0.33 | -2.682 | 0.195 | 0.082 | 0.086 | 2.877 |
| 0.34 | -2.851 | 0.19 | 0.078 | 0.08 | 3.041 |
| 0.35 | -3.025 | 0.182 | 0.076 | 0.089 | 3.207 |
| 0.36 | -3.204 | 0.175 | 0.087 | 0.085 | 3.379 |
| 0.37 | -3.39 | 0.166 | 0.085 | 0.08 | 3.556 |
| 0.38 | -3.581 | 0.158 | 0.078 | 0.076 | 3.739 |
| 0.39 | -3.779 | 0.168 | 0.071 | 0.072 | 3.947 |
| 0.4 | -3.983 | 0.162 | 0.064 | 0.067 | 4.145 |
| 0.41 | -4.194 | 0.156 | 0.065 | 0.063 | 4.35 |
| 0.42 | -4.413 | 0.149 | 0.053 | 0.072 | 4.562 |
| 0.43 | -4.639 | 0.149 | 0.053 | 0.07 | 4.788 |
| 0.44 | -4.873 | 0.144 | 0.05 | 0.067 | 5.017 |
| 0.45 | -5.116 | 0.139 | 0.04 | 0.065 | 5.255 |
| 0.46 | -5.367 | 0.133 | 0.04 | 0.062 | 5.5 |
| 0.47 | -5.628 | 0.128 | 0.034 | 0.059 | 5.756 |
| 0.48 | -5.9 | 0.123 | 0.035 | 0.03 | 6.023 |
| 0.49 | -6.181 | 0.118 | 0.025 | 0.028 | 6.299 |
| 0.5 | -6.475 | 0.114 | 0.024 | 0.026 | 6.589 |
| 0.51 | -6.78 | 0.109 | 0.021 | 0.024 | 6.889 |
| 0.52 | -7.097 | 0.105 | 0.022 | 0.021 | 7.202 |
| 0.53 | -7.429 | 0.103 | 0.029 | 0.019 | 7.532 |
| 0.54 | -7.775 | 0.099 | 0.024 | 0.016 | 7.874 |
| 0.55 | -8.136 | 0.096 | 0.02 | 0.013 | 8.232 |
| 0.56 | -8.513 | 0.092 | 0.02 | 0.011 | 8.605 |
| 0.57 | -8.908 | 0.088 | 0.018 | 0.01 | 8.996 |
| 0.58 | -9.322 | 0.066 | 0.01 | 0.008 | 9.388 |
| 0.59 | -9.756 | 0.063 | 0.006 | 0.006 | 9.819 |
| 0.6 | -10.212 | 0.059 | -0.005 | 0.004 | 10.271 |
| 0.61 | -10.691 | 0.055 | -0.012 | 0.002 | 10.746 |
| 0.62 | -11.195 | 0.051 | 0.001 | 0.013 | 11.246 |
| 0.63 | -11.727 | 0.053 | 0 | 0.012 | 11.78 |
| 0.64 | -12.288 | 0.05 | 0.005 | 0.011 | 12.338 |
| 0.65 | -12.881 | 0.047 | -0.004 | 0.01 | 12.928 |
| 0.66 | -13.509 | 0.044 | -0.004 | 0.009 | 13.553 |
| 0.67 | -14.176 | 0.041 | -0.003 | 0.008 | 14.217 |
| 0.68 | -14.883 | 0.018 | -0.003 | 0.006 | 14.901 |
| 0.69 | -15.637 | 0.016 | -0.007 | 0.006 | 15.653 |
| 0.7 | -16.441 | 0.013 | -0.011 | 0.005 | 16.454 |
| 0.71 | -17.3 | 0.009 | -0.002 | 0.004 | 17.309 |
| 0.72 | -18.22 | 0.033 | 0.006 | 0.003 | 18.253 |
| 0.73 | -19.209 | 0.032 | 0.01 | 0.002 | 19.241 |
| 0.74 | -20.274 | 0.031 | 0.007 | 0.01 | 20.305 |
| 0.75 | -21.424 | 0.03 | 0.006 | 0.01 | 21.454 |
| 0.76 | -22.669 | 0.023 | 0.011 | 0.01 | 22.692 |
| 0.77 | -24.024 | 0.022 | 0.01 | 0.009 | 24.046 |
| 0.78 | -25.501 | 0.021 | 0.007 | 0.009 | 25.522 |
| 0.79 | -27.119 | 0.019 | 0.005 | 0.008 | 27.138 |
| 0.8 | -28.898 | 0.004 | 0.004 | 0.008 | 28.902 |
